# Integrated care and interprofessional education

**DOI:** 10.1111/tct.13552

**Published:** 2022-11-16

**Authors:** Jill Thistlethwaite

**Affiliations:** ^1^ University of Technology Sydney Sydney New South Wales Australia

Once upon a time in a land far away from my current location, I worked as a general practitioner (GP) in a practice that for a few years provided what could be called *integrated care*. The practice staff did not use that term; we thought of ourselves as a primary care team aiming to provide the best possible care to patients and their community. It was a time in England in the 1990s when there was yet another National Health Service (NHS) transformation that gave some larger general practices their own budgets with full control over how these were spent. Funding became more transparent and allowed us to employ health and social care professionals, including nurses, physiotherapists, psychologists and a pharmacist, co‐located within the practice, benefiting both consumers and staff. We had regular meetings and practice‐wide interprofessional education (IPE). Patients accessed a wide range of services through one point of contact and, as GPs, we knew what was happening where and to whom. There were challenges and we had a lot to learn about contracts and costs, as purchasers of care from providers such as local hospitals and tertiary centres.

This fairy tale did not have a happy ending. The funding approach changed yet again, and services became fragmented as professionals moved to a district‐wide service rather than a practice‐based one: dis‐integrated care.

This experience taught me how the quality of the services we provide are vulnerable to systems change, over which care professionals have little control. Transformation is fancy terminology tending to emphasise how patient care will improve. But aspirational wording is easy to say but more difficult to enact. Outcomes tend to be measured through a medical and organisational gaze, ignoring what matters most to patients and with patchy collection of patient‐reported outcome measures (PROMs).

There are several definitions of the term ‘integrated care’. They emphasise seamless, effective and efficient care; coordination of structural links between services; the patient being in control or in partnership; cooperation amongst providers and removing barriers to care. Integration can be between primary and secondary care (community and hospital‐based services) and/or between health and social care. Of course, such care does exist and for some patients. However, what we experience, as consumers and professionals, frequently does not match up to the vision. Patient‐facing professionals tend to hear about and deal with problems rather than smooth integrated successes.

The *International Journal of Integrated Care* in its 20th anniversary issue included papers from countries with diverse health systems and varying levels of implementation of integration.^1^ Success was patchy in all jurisdictions, partly due to lack of consensus on the meaning of ‘integrated care’ and therefore its intended outcomes and how these might be measured. If the professionals cannot agree what integrated care should look like in practice, what sense can patients/consumers make of the term?

If the professionals cannot agree what integrated care should look like in practice, what sense can patients/consumers make of the term?

There are many reasons for disintegration of services: Lack of resources including sufficient professionals; funding models; poor communication amongst services; inexperience with collaboration; poor knowledge of the roles and responsibilities of other agencies and their personnel; and unfit for purpose electronic health and social care records hampering continuity of care. Enablers are adequate resourcing, shared values, effective communication, integrated IT systems, administrative flexibility, and appropriately trained clinicians.^2^

This brings me to education. In 2015, I was invited to speak about IPE at an interdisciplinary summit on global approaches to integrated health care convened by the American Psychological Society. The summit's recommendations are still relevant, emphasising the importance of interprofessional collaborative practice and communities to advocate for and build change, the need to implement IPE for integrated care and the importance of research and evaluation.^3^

For competency‐based education devotees, competency 1 of the World Health Organisation's 2022 framework for universal health coverage is ‘provides the best possible health care that supports an approach to health services that is effective, equitable, inclusive, integrated, people centred, safe and timely.’^4^ However, a major barrier to developing competencies for integrated care delivery is the lack of sufficient models to observe and be immersed in during education and training at all levels. In addition, where such models do exist, the level of integration may not be apparent to learners unless they are also involved in the patient experience as well as the professional one. While theory might be taught, in my experience experiential learning is less common. Some reflections on what is needed for integrated care education based on mine and others' experiences over 30 years are in Box 1.

A major barrier to developing competencies for integrated care delivery is the lack of sufficient models to observe and be immersed in during education and training.

Integrated care requires a consumer‐facing consensus definition, a commitment to IPE, facilitation of interprofessional collaborative practice through appropriate funding models and partnerships. Both health and social care services, and education provision need complementary change to realise the vision.

Integrated care requires a consumer‐facing consensus definition, a commitment to IPE, facilitation of interprofessional collaborative practice through appropriate funding models and partnerships.

**Box 1: IPE for integrated care delivery—elements required**

- High level support and sustained funding models for care delivery and training required^5^
- IPE along the continuum from pre‐qualification to continuing professional development
- The patient voice in planning and delivering education
- Case‐based learning
- Longitudinal clinical placements to enable integrated team membership
- IPE across pre‐qualification health and social care training
- Education about the importance of partnership working^6^
- Integration of learning across sectors through support of communities of practice^7^
- Technology‐enhanced learning^6^
- In‐service team‐based education with students as observers/participants
- Understanding/awareness of roles, responsibilities, scope of practice and boundaries
- Knowledge‐sharing culture^5^
- Workplace integrated learning for teams: informal and formal

## CONFLICT OF INTEREST

The authors have no conflict of interest to disclose.

## ETHICS STATEMENT

The authors have no ethical statement to declare.

## References

[1] International Journal of Integrated Care. 20^th^ anniversary issue. 2021. Available at: https://integratedcarefoundation.org/ijic-international-journal-integrated-care

[2] Coates D , Coppleson D , Travaglia J . Factors supporting the implementation of integrated care between physical and mental health services: an integrative review. J Interprof Care. 2022;36(2):245–58. 10.1080/13561820.2020.1862771 33438489

[3] Kearney LK , Zeiss AM , McCabe MA , Thistlethwaite JE , Chana N , Chen S , et al. Global approaches to integrated care: best practices and ongoing innovation. Am Psychol. 2020;75(5):668–82. 10.1037/amp0000490 31393143

[4] WHO . Global Competency and Outcomes Framework for Universal Health Coverage Geneva: World Health Organization; 2022 Licence: CC BY‐NC‐SA 3.0 IGO.

[5] Clouder L , Bluteau P , Jackson JA , Adefila A , Furlong J . Education for integrated working: a qualitative research study exploring and contextualizing how practitioners learn in practice. J Interprof Care. 2022;36(1):24–33. 10.1080/13561820.2021.1889485 34402733

[6] Howarth M , Holland K , Grant MJ . Education needs for integrated care: a literature review. J Adv Nurs. 2006;56(2):144–56. 10.1111/J.1365-2648.2006.03992.X 17018063

[7] Zhao QJ , Cupido N , Whitehead CR , Mylopoulos M . What role can education play in integrated care? Lessons from the ECHO (Extensions for Community Health Outcomes) concussion program. J Integr Care. 2022;30(4). 10.1108/JICA-01-2022-0012

